# Diagnosis of cognitive impairment and dementia: blood plasma and optical coherence tomography

**DOI:** 10.1093/braincomms/fcae472

**Published:** 2024-12-27

**Authors:** Vidishaa Jali, Qinglin Zhang, Joyce Ruifen Chong, Damon Wong, Bingyao Tan, Gerhard Garhöfer, Saima Hilal, Mitchell K P Lai, Leopold Schmetterer, Christopher Li-Hsian Chen, Jacqueline Chua

**Affiliations:** Department of Pharmacology, Memory Aging and Cognition Centre, Yong Loo Lin School of Medicine, National University of Singapore, Singapore 117600, Singapore; Department of Pharmacology, Memory Aging and Cognition Centre, Yong Loo Lin School of Medicine, National University of Singapore, Singapore 117600, Singapore; Department of Neurosurgery, Tsinghua University Yuquan Hospital, Beijing 100040, China; Department of Pharmacology, Memory Aging and Cognition Centre, Yong Loo Lin School of Medicine, National University of Singapore, Singapore 117600, Singapore; Singapore Eye Research Institute, Singapore National Eye Centre, The Academia, Singapore 169856, Singapore; Ophthalmology and Visual Sciences Academic Clinical Program, Duke-NUS Medical School, National University of Singapore, Singapore 169857, Singapore; SERI-NTU Advanced Ocular Engineering (STANCE), Nanyang Technological University, Singapore 639798, Singapore; School of Chemical and Biological Engineering, Nanyang Technological University, Singapore 637371, Singapore; Institute of Molecular and Clinical Ophthalmology, The University of Basel, Basel 4031, Switzerland; Singapore Eye Research Institute, Singapore National Eye Centre, The Academia, Singapore 169856, Singapore; Ophthalmology and Visual Sciences Academic Clinical Program, Duke-NUS Medical School, National University of Singapore, Singapore 169857, Singapore; SERI-NTU Advanced Ocular Engineering (STANCE), Nanyang Technological University, Singapore 639798, Singapore; School of Chemical and Biological Engineering, Nanyang Technological University, Singapore 637371, Singapore; Institute of Molecular and Clinical Ophthalmology, The University of Basel, Basel 4031, Switzerland; Department of Clinical Pharmacology, Medical University Vienna, Wien 1090, Austria; Saw Swee Hock School of Public Health, National University of Singapore, Singapore 117549, Singapore; Department of Pharmacology, Memory Aging and Cognition Centre, Yong Loo Lin School of Medicine, National University of Singapore, Singapore 117600, Singapore; Singapore Eye Research Institute, Singapore National Eye Centre, The Academia, Singapore 169856, Singapore; Ophthalmology and Visual Sciences Academic Clinical Program, Duke-NUS Medical School, National University of Singapore, Singapore 169857, Singapore; SERI-NTU Advanced Ocular Engineering (STANCE), Nanyang Technological University, Singapore 639798, Singapore; School of Chemical and Biological Engineering, Nanyang Technological University, Singapore 637371, Singapore; Institute of Molecular and Clinical Ophthalmology, The University of Basel, Basel 4031, Switzerland; Fondation Ophtalmologique Adolphe De Rothschild, Paris 75019, France; Center for Medical Physics and Biomedical Engineering, Medical University Vienna, Wien 1090, Austria; Department of Pharmacology, Memory Aging and Cognition Centre, Yong Loo Lin School of Medicine, National University of Singapore, Singapore 117600, Singapore; Singapore Eye Research Institute, Singapore National Eye Centre, The Academia, Singapore 169856, Singapore; Ophthalmology and Visual Sciences Academic Clinical Program, Duke-NUS Medical School, National University of Singapore, Singapore 169857, Singapore

**Keywords:** Alzheimer’s disease, retina, mild cognitive impairment, optical coherence tomography, blood plasma

## Abstract

Accurate and early diagnosis of Alzheimer's disease and vascular dementia is crucial for enabling timely interventions and improving patient outcomes. This study evaluates the diagnostic performance of plasma biomarkers (neurofilament light chain and phosphorylated tau181) and retinal biomarkers (retinal nerve fibre layer and ganglion cell-inner plexiform layer), individually and in combination, in differentiating moderate cognitive impairment and dementia from mild cognitive impairment and no cognitive impairment. A cross-sectional study was conducted involving 509 participants, aged 50 and older, recruited from a memory clinic. The participants were categorized as normal (*n* = 100), mild cognitive impairment (*n* = 144), moderate cognitive impairment (*n* = 90) or dementia (*n* = 175) based on detailed clinical assessments, neuropsychological testing and MRI scans. The thickness of the ganglion cell-inner plexiform layer (*P* < 0.001) and retinal nerve fibre layer (*P* = 0.030) decreased progressively from normal cognition to cognitive impairment and dementia. The thickest layers were observed in individuals with no cognitive impairment (mean ± standard deviation: ganglion cell-inner plexiform layer: 76 ± 11 µm, retinal nerve fibre layer: 92 ± 10 µm), while the thinnest layers were found in individuals with dementia (ganglion cell-inner plexiform layer: 72 ± 14 µm, retinal nerve fibre layer: 89 ± 12 µm). Plasma biomarker levels increased progressively from normal cognition to cognitive impairment and dementia (*P* < 0.001). Levels were lowest in individuals with no cognitive impairment [median (interquartile range): neurofilament light chain: 15 (9) pg/mL, phosphorylated tau181: 1.85 (1.00) pg/mL] and highest in those with dementia [neurofilament light chain: 34 (27) pg/mL, phosphorylated tau181: 3.24 (2.81) pg/mL]. After adjusting for retinal scan signal strength, neurofilament light chain showed a stronger negative association with retinal nerve fibre layer thickness [standardized beta estimate (*β*) = −0.184] and ganglion cell-inner plexiform layer thickness (*β* = −0.139) compared to phosphorylated tau181, which exhibited weaker associations with ganglion cell-inner plexiform layer (*β* = −0.091) and retinal nerve fibre layer (*β* = −0.059). While retinal parameters provided modest discriminatory ability (AUC = 0.60), plasma biomarkers demonstrated superior diagnostic performance (AUC = 0.76). Notably, neurofilament light chain had a stronger association with retinal thinning than phosphorylated tau181 and offered superior diagnostic value for identifying moderate cognitive decline. These findings underscore the potential of plasma biomarkers, particularly neurofilament light chain, for the early detection of dementia.

## Teaser

Jali *et al*. finds that the plasma neurofilament light chain, more strongly linked to retinal thinning, is a better biomarker than phosphorylated tau181 for differentiating moderate cognitive decline. This suggests plasma biomarkers, especially neurofilament light chain, could be valuable for early dementia detection.

## Graphical Abstract

**Graphical abstract:**
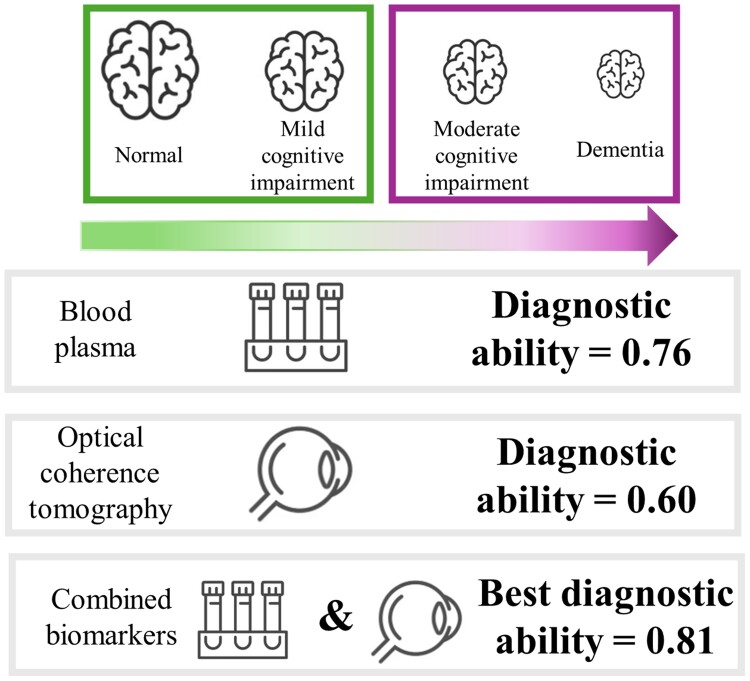

## Introduction

Dementia, encompassing both Alzheimer's disease and vascular dementia (VaD), is a progressive neurodegenerative disorder that compromises daily activities.^1^ Alzheimer’s disease is the leading cause of dementia globally.^2^ As populations age, the prevalence of dementia rises, presenting significant healthcare challenges.^3^ Individuals with moderate cognitive impairment without dementia (CIND) have a greater risk of dementia progression than those with mild CIND.^4^ An early and accurate diagnosis of moderate CIND and dementia is crucial as it allows for prompt interventions such as lifestyle changes and medications, potentially slowing disease progression and enhancing quality of life.^5^

Traditional diagnostic methods for dementia, including clinical assessments, neuropsychological testing and brain imaging (MRI, CT), have significant limitations, including the necessity for specialized expertise, potential subjectivity in assessments, and the inability to detect early-stage cognitive decline. Moreover, these methods can be costly, and time-consuming. This highlights the need for objective and accessible diagnostic tools^6^ such as optical coherence tomography (OCT)^7^ and blood plasma biomarkers.^8^ OCT, a non-invasive imaging technique, shows promise for early detection of dementia.^7^ It enables high-resolution imaging of the retina, a neural extension of the central nervous system.^9^ The retina, sharing anatomical and physiological similarities with the brain, undergoes neurodegenerative changes in Alzheimer’s disease.^10^ A meta-analysis of 1257 Alzheimer’s disease patients, 305 mild cognitive impairment (MCI) patients, and 1460 controls revealed thinner retinal nerve fibre layer (RNFL) and ganglion cell-inner plexiform layer (GCIPL), compared to cognitively normal controls.^11^ Next, blood biomarkers may be reflective of the pathophysiological changes implicated in dementia.^8^ For example, plasma neurofilament light chain (NfL) and phosphorylated tau181 (P-tau181) are associated with neurodegeneration and brain amyloid burden, respectively.^12^

Despite advancements in OCT and plasma biomarker analysis, their combined effectiveness in the early detection of high-risk individuals for moderate CIND and dementia remains unclear. This is clinically relevant, as moderate CIND is a stronger predictor of dementia than mild CIND.^4^ This study addresses this gap by investigating the association of OCT parameters and plasma biomarkers and the diagnostic accuracy of OCT parameters and plasma biomarkers in a cohort of individuals aged 50 years and above enrolled in a memory clinic. Understanding how these biomarkers relate to each other and how they can be used together for early detection of moderate CIND and dementia is crucial. This knowledge can pave the way for more sensitive and effective diagnostic strategies, improving clinical outcomes.

## Materials and methods

### Study population

This cross-sectional study included participants aged 50 years and above enrolled in a memory clinic from January 2010 to February 2020. Neurologists, psychologists and researchers determined diagnoses by consensus, reviewing clinical assessments, blood tests, neuropsychological tests and MRI scans. Neuropsychological domain impairments and diagnoses were based on the vascular dementia battery, which assesses six cognitive domains: attention, language, verbal and visual memory, visuoconstruction and visuomotor speed. The vascular dementia battery has been validated in a Singaporean sample of community-dwelling controls and dementia patients.^13^ Continuous scoring is used for all tests, and passing scores are adjusted based on factors like age and education.^14^ Participants failing more than half of the tests in a vascular dementia battery domain were considered impaired. Those with at least one impairment but not meeting dementia criteria were classified as CIND: mild (1–2 impairments) or moderate (3+ impairments). Cognitively unimpaired [no cognitive impairment (NCI)] participants were recruited from the Epidemiology of Dementia in Singapore study,^15^ having no objective cognitive impairment or functional decline.^16^ Individuals aged 60 and above who screened positive for cognitive impairment on the Abbreviated Mental Test and self-reported decline in the Singapore Epidemiology of Eye Diseases population-based study were invited to Epidemiology of Dementia in Singapore. Those without cognitive impairment after neuropsychological testing were classified as NCI. Dementia diagnoses were made according to the Diagnostic and Statistical Manual, 4th edition criteria.^17^ This study was approved by the National Healthcare Group Domain-Specific Review Board (NHG DSRB reference number 2018/01098 and 2010/00017) and adhered to the Declaration of Helsinki. All participants or their primary caregivers gave written informed consent.

Participants were excluded if they had hypoxic, anoxic, hypotensive, hypertensive, uremic or hepatic encephalopathy; traumatic, nutritional or toxic CNS disorders; substance abuse affecting the CNS; intracerebral haemorrhage; cranial arteritis; CNS inflammatory vasculitis; moyamoya disease; CNS infection; space-occupying intracranial mass lesion; obstructive or normal pressure hydrocephalus; uncontrolled epilepsy; medical illness requiring corticosteroids or immunosuppressants; a moribund state; or significant aphasia or dysarthria.

We excluded patients with glaucoma to mitigate the risk of angle-closure glaucoma induced by pharmacological dilation, but not those with age-related macular degeneration (AMD) and diabetic retinopathy. Excluding patients with AMD and diabetic retinopathy could introduce selection bias, as these conditions are often associated with age-related cognitive decline.^18^ Medical histories (diabetes, hypertension) were collected, and seated blood pressure was measured using an automated device during their clinical visits.

### OCT imaging

**Figure 1:**
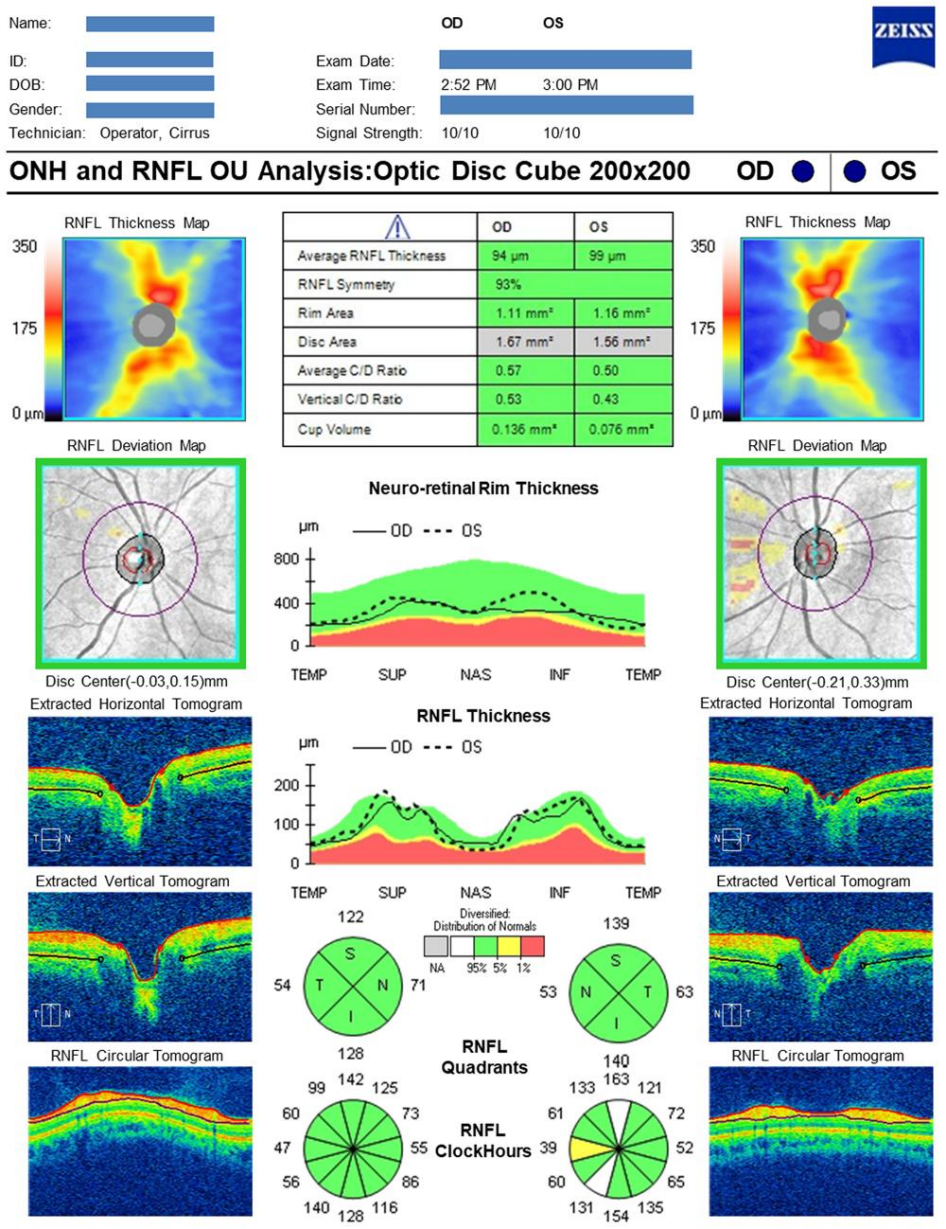
**Representative optic nerve head and RNFL OCT analysis of a cognitively normal individual**. The RNFL thickness quadrants colour code map shows green, indicating RNFL thickness within normal limits in all four sectors. C/D, cup/disc; ID, identification; INF, inferior; NAS, nasal; OD, right eye; OS, left eye; OU, both eyes; TEMP, temporal; SUP, superior; NAS, nasal; INF, inferior; µm, micrometre. The statistical analysis underlying the colour-coded map was performed directly by the commercial device manufacturer and is not publicly available.

**Figure 2:**
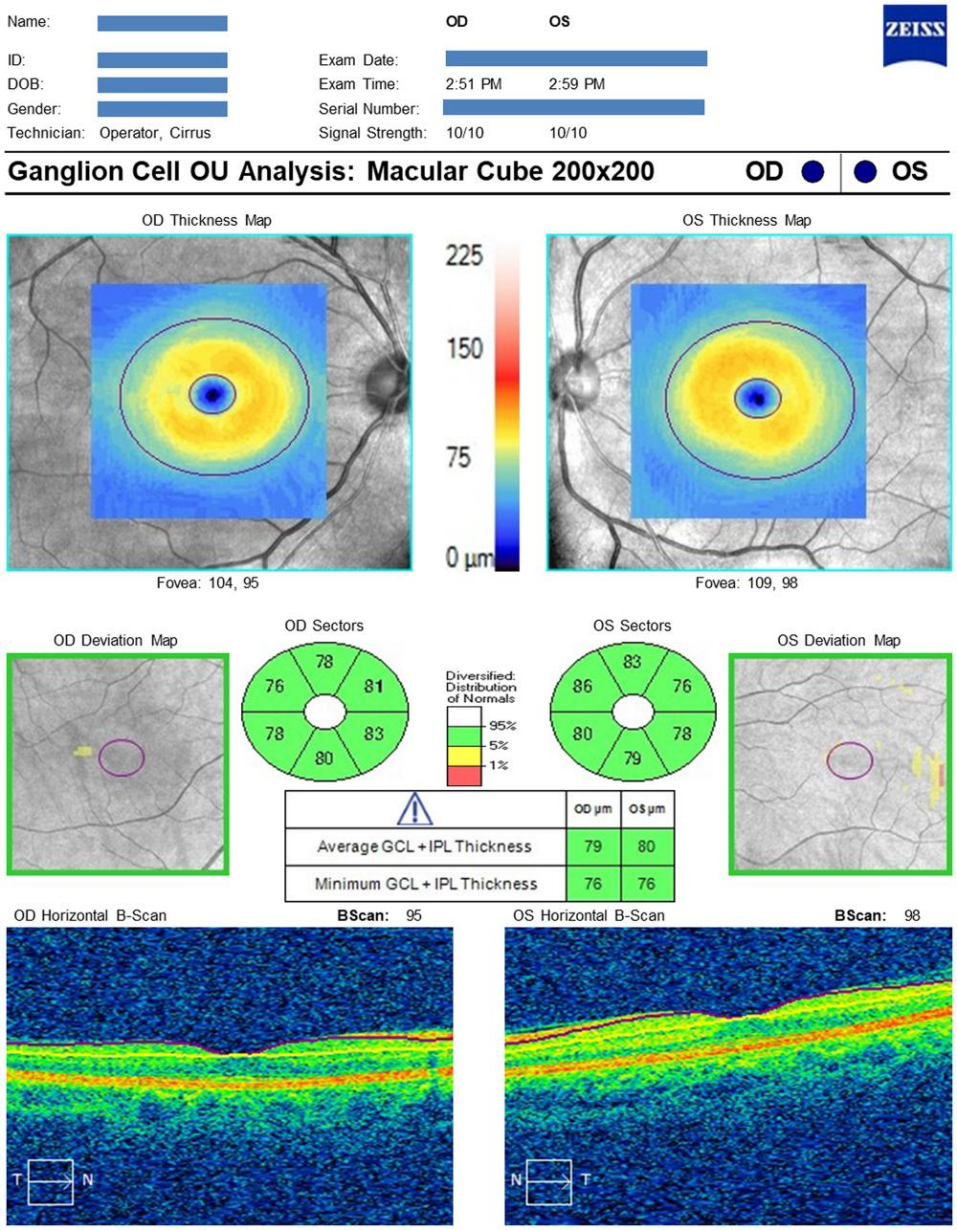
**Representative GCIPL OCT analysis of a cognitively normal individual**. The GCIPL thickness sectors colour code map shows green, indicating GCIPL thickness within normal limits in all six sectors. Bscan, brightness scan; ID, identification; INF, inferior; NAS, nasal; OD, right eye; OS, left eye; OU, both eyes; µm, micrometre. The statistical analysis underlying the colour-coded map was performed directly by the commercial device manufacturer and is not publicly available.

All participants underwent OCT imaging using the Cirrus spectral domain-OCT (Carl Zeiss Meditec, Inc., Dublin, CA, USA).^19^ Two scan protocols [optic disc (Fig. 1) and macula (Fig. 2)] were acquired. One trained grader, masked to participant characteristics, reviewed OCT datasets, excluding those with poor quality (scan signal strength <6 and/or excessive movement artefacts and/or inconsistent signal intensity across the scan and/or segmentation failure) or missing variables.^20^ Only data from one eye per participant were used. The right eye was used if both eyes met the eligibility criteria described, but the left eye was used if only left eye data were available. Circumpapillary RNFL and GCIPL thickness measurements were extracted from Cirrus Review Software (software version 11.0.0.29946).

### Blood plasma biomarkers

Non-fasting blood was collected into ethylenediaminetetraacetic acid tubes, centrifuged at 2000 × *g* for 10 min at 4°C, aliquoted into polypropylene tubes, and stored at −80°C until analysis. Plasma P-tau181 and NfL levels were measured using Simoa HD-1 analyzer using commercially available Simoa pTau-181 Advantage V2 kit and Simoa NF-light Advantage kit, respectively (Quanterix, Billerica, MA, USA).

### Statistical analysis

Statistical analyses were performed using Statistical Package for Social Science version 29.0.1.0 (IBM, Armonk, NY, USA). One-way analysis of variance was used for continuous variables and Chi-square tests were used for categorical variables. Characteristics were summarized as mean ± standard deviation (SD) or numbers/percentages.

*P* trend test was used to evaluate if there was a linear trend in OCT thickness parameters and blood plasma biomarker levels across the four categories of cognitive impairment, ordered from NCI to mild CIND, moderate CIND and dementia. A linear trend indicates a steady increase or decrease in a variable across the categories, without abrupt changes. As plasma NfL and P-tau181 distribution was skewed, log transformation of the variable was applied to all the statistical tests, and values were summarized as median (interquartile range).

The associations between OCT parameters and plasma biomarkers were investigated using linear regression with log-transformed values for plasma biomarkers as outcomes. The model was adjusted for OCT scan signal strength.^21^ Standardized beta coefficients were reported to allow for a direct comparison of the relative importance of the OCT and plasma biomarkers.

Diagnostic accuracies were assessed using the area under the receiver operating characteristic curve (AUC) to evaluate the ability of each parameter to differentiate between diseased (moderate CIND, dementia) and non-diseased (NCI, mild CIND) populations, adjusted for OCT scan signal strength. Hypertension is linked to both dementia^22^ and reduced RNFL thickness^23-25^ Adjusting for hypertension could mask the true relationship between retinal changes and cognitive decline. To capture the total effect, we did not adjust for hypertension. Both single predictors and combinations of predictors (e.g. blood plasma biomarkers and OCT measurements) were considered to determine whether combining predictors improved discrimination. The receiver operating characteristic curve procedure in SPSS allows for direct input of multiple predictors, eliminating the need for a two-step process (IBM Corp. Armonk, NY, USA: IBM Corp). AUCs were compared between single-predictor models and combined-predictor models using the likelihood ratio test (RStudio, PBC, Boston, MA, USA), which is better suited for comparing nested receiver operating characteristic curve models.^26^ To avoid the accumulation of Type I error due to multiple comparisons between OCT parameters, plasma biomarkers and clinical factors, we employed a conservative Bonferroni correction and considered results statistically significant at the level of *α* = 0.05/3 = 0.017. For other analyses, *P* < 0.05 was considered significant.

## Results

Out of the 648 participants enrolled, 139 were excluded because of poor-quality OCT scans (*n* = 43) and missing plasma biomarker data (*n* = 96; Supplementary Fig. 1). The remaining 509 participants (100 NCI, 144 mild CIND, 90 moderate CIND and 175 dementia) were included in the analysis. Of the remaining participants, 431 had RNFL data (89 NCI, 136 mild CIND, 75 moderate CIND and 131 dementia) and were included in the plasma and RNFL association analysis. Meanwhile, 500 had GCIPL data (98 NCI, 140 mild CIND, 89 moderate CIND and 173 dementia) and were included in the plasma and GCIPL association analysis. For the diagnostic analysis, we excluded 87 participants who had only RNFL or GCIPL data, which left 422 participants with both GCIPL and RNFL data (87 NCI, 132 mild CIND, 74 moderate CIND and 129 dementia).

Table 1 showed that CIND and dementia were older (*P* < 0.001), more likely to be an ApoE carrier (*P* = 0.001), had fewer years of education (*P* < 0.001), higher systolic blood pressure (*P* = 0.045, and higher prevalence of hypertension (*P* < 0.001) and diabetes (*P* = 0.002) compared to the NCI group.

**Table 1:** Characteristics of the participants included in the study

Characteristics	No cognitive impairment (*n* = 100)	Mild CIND (*n* = 144)	Moderate CIND (*n* = 90)	Dementia (*n* = 175)	*P*-value
Age	69 ± 8	72 ± 7	75 ± 7	75 ± 7	**<0**.**001**
Males (%)	45 (45%)	76 (53%)	38 (42%)	66 (38%)	0.059
ApoE 4 carrier status	13 (13%)	49 (34%)	32 (36%)	53 (30%)	**0**.**001**
Total number of years of education	10 ± 5	8 ± 5	7 ± 5	5 ± 4	**<0**.**001**
Smoking status—ever smoker	21 (21%)	34 (24%)	26 (29%)	47 (27%)	0.565
Body mass index (kg/m^2^)	24 ± 4	24 ± 5	24 ± 4	24 ± 4	0.696
Systolic blood pressure (mmHg)	138 ± 18	143 ± 21	139 ± 23	144 ± 21	**0**.**045**
Diastolic blood pressure (mmHg)	73 ± 11	75 ± 12	71 ± 12	72 ± 11	0.069
Hypertension	58 (58%)	93 (65%)	67 (74%)	142 (81%)	**<0**.**001**
Diabetes	20 (20%)	49 (34%)	35 (39%)	74 (42%)	**0**.**002**
Hyperlipidaemia	67 (67%)	104 (72%)	70 (78%)	128 (73%)	0.421
Age-related macular degeneration	54 (54%)	83 (58%)	49 (54%)	81 (46%)	0.216
Diabetic retinopathy	5 (5%)	6 (4%)	5 (6%)	19 (11%)	0.079

CIND, cognitive impairment no dementia.

*P*-values were calculated using one-way analysis of variance for continuous variables and the chi-square test for categorical variables.

Significant *P*-values (<0.05) are indicated in bold.

### Distributions of OCT parameters and blood plasma biomarkers

**Figure 3:**
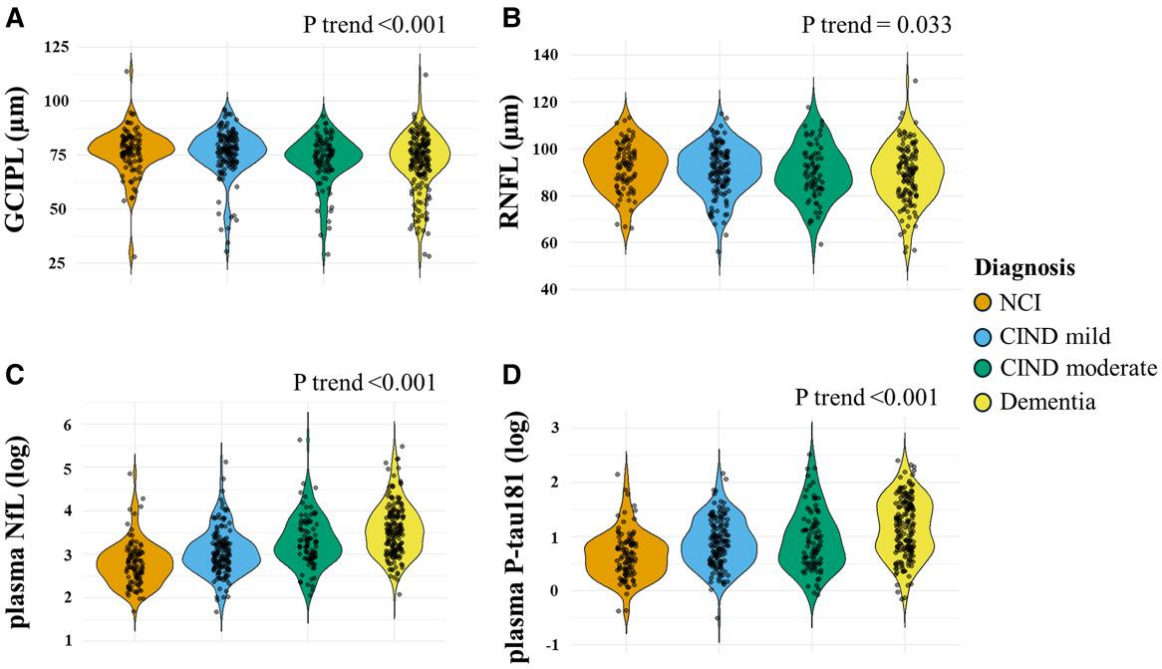
**Distribution of OCT and plasma biomarkers across neurodegenerative disease stages.** Violin plots show the distribution of OCT biomarkers [(**A**) GCIPL and (**B**) RNFL] and plasma biomarkers [(**C**) neurofilament light chain (NfL, log-transformed) and (**D**) phosphorylated tau181 (P-tau181, log-transformed)] across disease stages. The colours represent different diagnostic groups: no cognitive impairment (NCI; orange), mild cognitive impairment no dementia (CIND mild; blue), moderate CIND (green) and dementia (yellow). Each data point represents a single measurement of the biomarker for an individual participant, and the violin shapes illustrate the density distribution of these values at each disease stage. As disease severity increases, GCIPL and RNFL thickness decrease, while NfL and P-tau181 levels increase. The ‘*P* trend’ values for all four biomarkers are significant (*P* < 0.001 for GCIPL, NfL and P-tau181; *P* = 0.033 for RNFL), indicating a significant linear relationship between the severity of cognitive impairment (NCI, mild CIND, moderate CIND and dementia) and these biomarkers. The *P* trend test was used to evaluate this relationship. For the RNFL and plasma biomarker analyses, data from *N* = 431 participants were included, while for GCIPL and plasma analyses, *N* = 500 participants were analysed.

The distributions of RNFL, GCIPL, plasma NfL and plasma P-tau181 across the groups (NCI, CIND mild, CIND moderate and dementia) are presented in Table 2 and Fig. 3. GCIPL and RNFL thickness decreased from normal cognition to CIND to dementia (*P* trend < 0.001 and *P* trend = 0.030, respectively), where it was thickest in NCI (mean ± SD: GCIPL: 76 ± 11 µm and RNFL: 92 ± 10 µm) and thinnest in individuals with dementia (mean ± SD: GCIPL: 72 ± 14 µm and RNFL: 89 ± 12 µm). Plasma NfL and P-tau181 levels increased from normal cognition to CIND to dementia (*P* trend < 0.001), where it was lowest in NCI [median (interquartile range): plasma NfL: 15 (9) pg/mL and plasma P-tau181: 1.85 (1.00) pg/mL] and highest in individuals with dementia [median (interquartile range): plasma NfL: 34 (27) pg/mL and plasma P-tau181: 3.24 (2.81) pg/mL]. As Fig. 3 illustrates, while several OCT measures exhibited statistically significant trends with cognitive status, the magnitude of the association was stronger for plasma biomarkers.

**Table 2:** Distributions of the GCIPL and RNFL thickness measurements with plasma NfL and *P*-tau181 levels

Characteristics	NCI	Mild CIND	Moderate CIND	Dementia	*P* trend
GCIPL (µm)	76 ± 11	76 ± 11	73 ± 12	72 ± 14	**<0**.**001**
RNFL (µm)	92 ± 10	91 ± 11	90 ± 12	89 ± 12	**0**.**033**
Plasma NfL (pg/mL)	15 (9)	20 (12)	25 (22)	34 (27)	**<0**.**001**
Plasma *P*-tau181 (pg/mL)	1.85 (1.00)	2.37 (1.61)	2.35 (1.84)	3.24 (2.81)	**<0**.**001**

GCIPL, ganglion cell-inner plexiform layer; NfL, Neurofilament light chain; CIND, cognitive impairment no dementia; P tau 181, phosphorylated tau at serine 181; RNFL, retinal nerve fibre layer.

Values are presented as mean ± standard deviation or median (interquartile range). The values presented are raw plasma biomarker values.

The *P* trend test was used to assess whether there was a gradual change (increasing or decreasing trend) in these measures across the groups (NCI, CIND mild, CIND moderate, and dementia).

Significant *P*-values (<0.05) are indicated in bold.

### Associations of OCT parameters and blood plasma biomarkers

**Figure 4:**
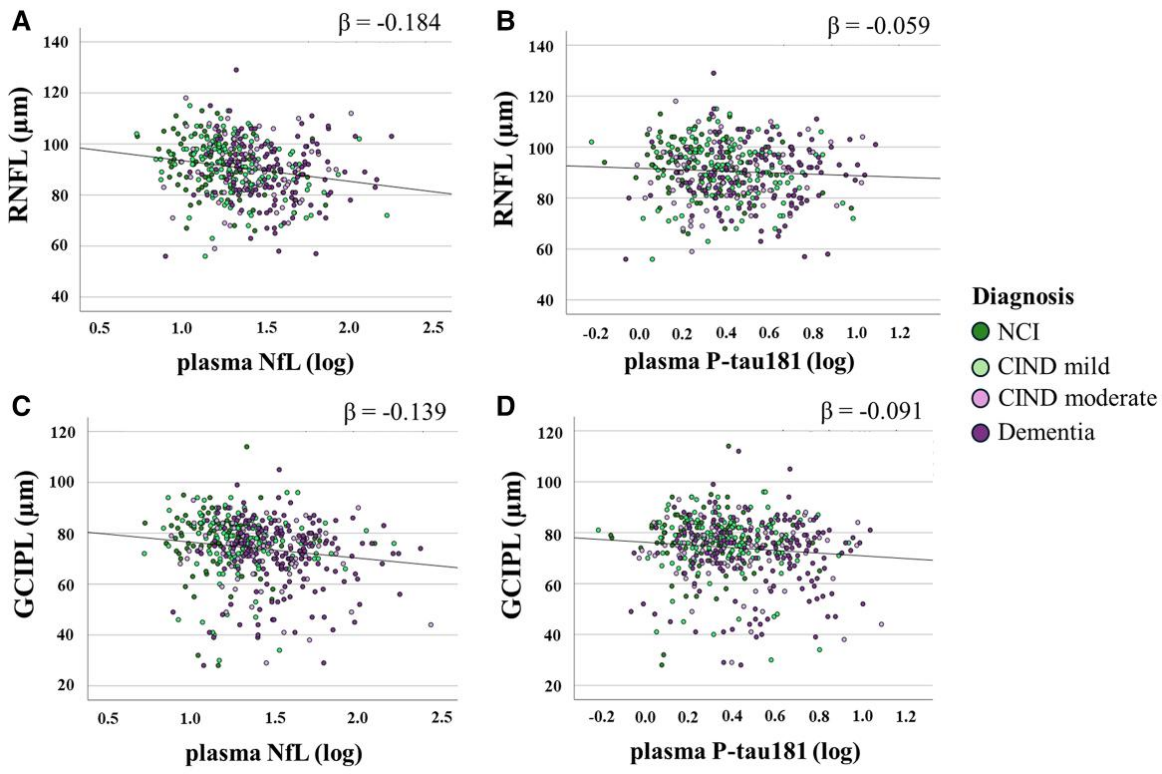
**Association of OCT parameters with plasma biomarkers.** Scatterplots show the relationship between RNFL (**A** and **B**) and GCIPL (**C** and **D**) thickness measurements with plasma neurofilament light chain (NfL; **A** and **C**) and phosphorylated tau181 (P-tau181; **B** and **D**) in patients with no cognitive impairment (NCI; green colour), mild cognitive impairment no dementia (CIND; light green colour), moderate CIND (light magenta colour) and dementia (magenta colour). Each data point represents a pair of measurements (one OCT measurement and one plasma biomarker measurement) from a single patient. The straight lines represent the linear relationship between the variables, using the ordinary least squares method. The associations between OCT parameters and plasma biomarkers were investigated using linear regression with log-transformed values for plasma biomarkers as outcomes and adjusted for OCT scan signal strength. The standardized beta estimates for NfL showed a stronger negative association with RNFL (*β* = −0.184, *P* < 0.001) and GCIPL (*β* = −0.139, *P* < 0.001) compared to P-tau181, which had weaker associations with GCIPL (*β* = −0.091, *P* = 0.027) and RNFL (*β* = −0.059, *P* = 0.045). *N* = 431 participants were used for the RNFL and plasma analyses and *N* = 500 participants for GCIPL and plasma analyses.

Figure 4 shows the scatterplots between GCIPL, RNFL, plasma NfL and plasma P-tau181. After controlling for the effects of OCT scan signal strength, the absolute values of the standardized beta estimates are relatively small, indicating weak relationships between the biomarkers. When comparing the magnitude of the standardized beta estimates, NfL demonstrated a slightly stronger negative association with both RNFL [standardized beta estimate (*β*) = −0.184] and GCIPL (*β* = −0.139) compared to P-tau181 which has weaker associations with GCIPL (*β* = −0.091) and RNFL (*β* = −0.059). For NfL associations, the standardized beta estimate for RNFL is −0.184, indicating that for every 1 SD decrease in RNFL thickness, there is a corresponding 0.184 SD increase in NfL levels, consistent with the observation of thinner RNFL and higher NfL levels in individuals with increasing cognitive decline.

### Diagnostic performance of OCT, blood plasma and clinical status to distinguish between moderate CIND/dementia (high risk) from NCI/mild CIND (low risk)

The diagnostic analysis of different factors, including plasma biomarkers (NfL and P-tau181), OCT parameters (RNFL and GCIPL) and clinical factors (age, years of education) to discriminate moderate CIND/dementia (high risk of cognitive decline) from NCI/mild CIND participants (low risk of cognitive decline), are shown in Table 3. The GCIPL (AUC = 0.60) outperformed RNFL (AUC = 0.56) when discriminating between the two groups (*P* < 0.001). Plasma NfL significantly outperformed *P*-tau181 in identifying the moderate CIND/dementia participants (AUC = 0.75 versus 0.65; *P* < 0.001).

**Table 3:** Diagnostic performance for discriminating moderate CIND/dementia from mild CIND/NCI individuals

	Area under ROC	95% Confidence interval	Sensitivity (%) at 80% specificity	*P*-value	*P*-value	*P*-value
OCT						
GCIPL	0.60	0.54–0.65	32%	Reference		
RNFL	0.56	0.50–0.61	29%	**<0.001**		
Combined OCT	0.60	0.55–0.65	36%			**<0**.**001**
Plasma						
NfL	0.75	0.70–0.80	58%		Reference	
P-tau181	0.65	0.60–0.71	45%		**<0.001**	
Combined plasma	0.76	0.71–0.80	60%			**<0**.**001**
Clinical status						
Age	0.66	0.61–0.72	42%			
Educational levels	0.70	0.66–0.70	46%			
Combined clinical status	0.74	0.70–0.79	52%			**0**.**001**
Combined models						
Combined OCT + Combined plasma + Combined clinical status	0.81	0.77–0.85	64%			**Reference**
Combined plasma + Combined clinical status	0.81	0.76–0.85	65%			0.061
NfL + Combined clinical status	0.80	0.75–0.84	62%			**0**.**011**
Combined OCT + Combined clinical status	0.75	0.71–0.80	52%			**<0**.**001**

GCIPL, ganglion cell-inner plexiform layer; NCI, no cognitive impairment; NfL, Neurofilament light chain; CIND, cognitive impairment no dementia; P tau 181, phosphorylated tau at serine 181; RNFL, retinal nerve fibre layer; ROC, receiver operating characteristic curve.

*P*-value indicates the paired comparisons with the reference parameter using the likelihood ratio test.

Significant *P*-values (<0.017 after Bonferroni correction) are indicated in bold.

**Figure 5:**
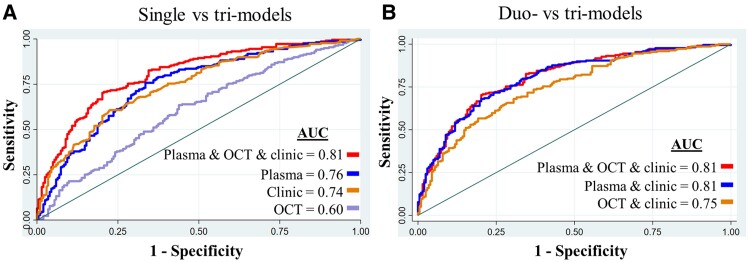
**Predictive power of tri-model versus single factors for differentiating cognitive impairment stages.** Receiver operating characteristic curves and corresponding AUC values comparing the predictive power of a tri-model (combining plasma biomarkers, OCT parameters, and clinical factors) to single factors and duo models in differentiating moderate CIND/dementia from NCI/mild CIND. Each point on the receiver operating characteristic curve represents a specific threshold for a diagnostic test. A multi-factor approach, combining OCT, plasma, and clinical data (AUC of 0.81; *P* = 0.001), significantly improved the accuracy compared to single factors (**A**). While OCT added value, plasma and clinical data (AUC of 0.81; *P* = 0.061) appeared to be essential components in these predictions (**B**). *N* = 422 participants were used for the diagnostic analysis.

The tri-model (combining OCT + plasma + clinical; AUC = 0.81) significantly outperformed single factors (*P* = 0.001), indicating that a multi-factor approach provides a more accurate prediction of moderate CIND/dementia from NCI/mild CIND (Fig. 5A). However, the tri-model performed similarly to the duo-model (plasma + clinical) in discriminating between moderate CIND/dementia and NCI/mild CIND (AUC = 0.81; *P* = 0.061; Fig. 5B). This suggests that while OCT adds value, plasma and clinical data might be sufficient for this specific task. The tri-model also outperformed these duo models [NfL only + clinical model (AUC = 0.80; *P* = 0.011) and OCT + clinical model (AUC = 0.75; *P* < 0.001)].

To assess the potential influence of ocular disorders on OCT measurements, we conducted sensitivity analyses excluding participants with AMD and diabetic retinopathy. Of the 509 participants, 278 (55%) had ocular disorders. Among the 231 participants without eye disease, 227 had available GCIPL data, and 196 had available RNFL data. Consequently, sensitivity analysis was performed on 192 participants with normal eyes (Supplementary Table 1). We found that the tri-model (combining OCT + plasma + clinical; AUC = 0.81) significantly outperformed single factors (*P* = 0.001) but performed similarly to the duo-model (plasma + clinical) in discriminating between moderate CIND/dementia and NCI/mild CIND (AUC = 0.79; *P* = 0.029, not significant after Bonferroni adjustment). The tri-model also outperformed these duo models: NfL only + clinical model (AUC = 0.78; *P* = 0.012) and the OCT + clinical model (AUC = 0.75; *P* < 0.001).

## Discussion

This cross-sectional study investigated the relationship between OCT parameters, plasma biomarkers and their diagnostic accuracy for moderate CIND and dementia. We found that GCIPL and RNFL thicknesses decreased from NCI to CIND to dementia, and plasma NfL and P-tau181 levels increased from NCI to CIND to dementia. While both GCIPL and RNFL are significantly correlated with NfL, only GCIPL showed a significant association with P-tau181, a marker specific to Alzheimer's disease. This suggests GCIPL thickness might be a more specific indicator of Alzheimer's compared to RNFL. The tri-model, combining GCIPL, plasma NfL and clinical factors, demonstrated the highest discrimination for moderate CIND and dementia, which translates to 66% sensitivity. However, the main driver of this discrimination was the inclusion of plasma biomarkers rather than OCT parameters. The findings suggest that while OCT parameters such as GCIPL show significant associations with plasma P-tau181, their contribution to diagnostic accuracy is marginal compared to plasma biomarkers. These results highlight the importance of incorporating plasma biomarkers into the diagnostic models for moderate CIND and dementia.

### Relationship between OCT and blood plasma biomarkers

Our study demonstrates a more pronounced association between the thinning of the RNFL and GCIPL with elevated plasma NfL levels compared to P-tau181 in patients with CIND and dementia. This finding aligns with the well-recognized role of NfL as a marker of neurodegeneration and axonal damage in Alzheimer’s disease and MCI.^27^ The association between OCT parameters and plasma NfL suggests that retinal changes captured by OCT may reflect underlying neurodegenerative processes in the brain. This suggests OCT could be a non-invasive tool for monitoring neurodegeneration in dementia and CIND.

Our findings are consistent with a large-scale German study linking inner retinal layers (GCIPL and RNFL) and plasma NfL.^28^ The consistency of our findings with this large-scale study adds further support to the potential clinical utility of OCT in evaluating neurodegenerative changes in dementia and MCI.

Comparing the magnitudes of the standardized beta estimates suggests that P-tau181 has a stronger association with GCIPL (*β* = −0.091) than with RNFL (*β* = −0.059). This finding suggests that changes in P-tau181, a well-established biomarker for Alzheimer's disease, are more closely linked to alterations in GCIPL than in RNFL. Mounting evidence suggests that morphological changes, like dendritic shrinkage, begin in neurons throughout the nervous system, including the retina, in neurodegenerative diseases.^29^ Similar to findings in mouse models of Alzheimer’s disease, retinal ganglion cell dendritic changes might occur before axonal degeneration.^30^ This earlier vulnerability of the GCIPL could explain why reductions in its thickness might serve as a potentially more sensitive marker for cognitive decline compared to the RNFL, which reflects damage to downstream axons.

### Value of OCT biomarkers in CIND and dementia detection

While traditional methods for diagnosing CIND and dementia, such as clinical assessments, cognitive tests and neuroimaging techniques,^6^ have been the mainstay, they are not without limitations. Neuroimaging techniques can be invasive and pose significant challenges for elderly individuals with cognitive issues. In contrast, OCT imaging is easier to perform and more cost-effective. Previous meta-analysis has suggested that OCT biomarkers, such as thinner GCIPL and RNFL, compared to healthy controls,^11^ could be valuable for high-risk moderate CIND and dementia screening.^7^ In the current study, GCIPL and RNFL thickness decreased consistently from normal cognition to MCI to dementia, but this decrease was minimal (3–4 µm between the normal cognition and dementia group). This indicates why OCT parameters showed limited effectiveness in distinguishing high-risk moderate CIND and dementia. Indeed, a recent meta-analysis reported a modest diagnostic performance (AUC = 0.70; 95% CI 0.53–0.79) of RNFL thickness for detecting Alzheimer’s disease compared with healthy controls.^31^

There are two potential explanations for the modest diagnostic performance of OCT parameters. First, RNFL thickness naturally varies between individuals due to eye shape and other anatomical features.^32-34^ This inherent variability can make it challenging to detect subtle group differences, particularly without accounting for anatomical factors. In our previous work,^35^ we adjusted anatomical factors in RNFL measurements, leading to improved differentiation between the normal cognition and MCI/Alzheimer’s disease groups. However, in this study, we did not employ these adjustments due to the limitations of the compensation model. This model requires both optic disc and macular scans, refractive error data, and is currently available only for Chinese, Malays and Indians. As a result, 14% of the participants could not be included in the model. We are actively developing a new model that does not require refractive error data and are working towards creating a unified model for Asian populations. Second, the current study included participants with a variety of eye diseases, whereas our earlier study did not.^35^ While we performed a quality check on the OCT scans to minimize segmentation errors, these conditions might have affected RNFL thickness in some individuals, potentially masking group differences between the diagnostic groups. Given that over half of our older adults had at least one eye condition, excluding data from individuals with eye condition could significantly reduce the sample size.^36^ The findings of the subgroup analysis of 192 participants with normal eyes were consistent with the results from the entire study population, demonstrating the robustness of our initial conclusions regarding the diagnostic potential of plasma in combination with clinical factors for distinguishing moderate CIND/dementia from NCI/mild CIND. Although the results were consistent across both the subgroup and the overall population, it is important to acknowledge the small sample size of the subgroup with non-diseased eyes, which may limit the generalizability of these findings.

The modest diagnostic performance observed for OCT in our study does not diminish their potential utility in dementia investigation. Research in glaucoma using OCT has shown that while the accuracy in cross-sectional studies may be limited due to variations among normal individuals, tracking changes in OCT parameters over time within the same individual can be valuable for detection.^37^ OCT parameters may hold promise as monitoring biomarkers for dementia. Therefore, longitudinal studies focusing on the development of dementia and CIND might offer improved diagnostic accuracy compared to cross-sectional approaches.

Future studies could enhance the diagnostic capability of OCT parameters by employing several strategies. These include using anatomically corrected scans to reduce the variability of retinal thickness measurements among normal individuals,^35^ separating vascular components from RNFL thickness measurements to enhance the accuracy,^25^ integrating OCT angiography imaging modality to capture both structural and vascular information of the retina^38-42^ and employing artificial intelligence-based algorithms.^43-45^ We primarily focused on comparing moderate CIND and dementia versus NCI and mild CIND, as this directly assesses the ability of predictors to differentiate between individuals with mild cognitive impairment and those with more severe cognitive decline. While not explored here, future studies with larger samples could analyse NCI versus all CIND/Dementia.

### Value of blood plasma biomarkers in high-risk moderate CIND and dementia detection

In this study, we affirm the clinical utility of blood plasma biomarkers NfL and P-tau181 for discriminating individuals with moderate CIND and dementia. We found that their plasma NfL and P-tau181 levels were increased, which differentiated participants with moderate CIND/dementia from NCI/mild CIND with high accuracy. Elevated NfL levels are a non-specific biomarker of neuroaxonal injury 17, while increased P-tau181 levels are associated with brain amyloid and tau burden, characteristic features of Alzheimer’s disease.^46^ These blood plasma biomarkers serve as important indicators of disease status and could help in the early detection of CIND/dementia.

### Strengths and limitations

The strengths of this study included the comprehensive phenotyping of participants, thorough neurocognitive assessments, and the use of OCT scans with quality assessment. Our study focused on two circulating biomarkers in a well-annotated cohort, allowing for head-to-head comparisons of various factors. These strengths enhance the robustness of our findings and the depth of our analysis. However, our study also has limitations. The cross-sectional design limited our ability to establish causality, and longitudinal studies will be required to confirm the predictive value of OCT parameters and blood plasma biomarkers for disease progression. Additionally, while we controlled for OCT signal scan quality and years of education, other unmeasured variables could have influenced our results. A potential limitation of our study is the possibility of selection bias due to missing OCT data. Individuals with more severe retinal atrophy may have been more likely to have poor-quality scans, leading to missing data. Future studies should consider strategies to minimize missing data, such as using an ultrafast OCT system with optimized post-processing image protocols.^47^

## Conclusions

We observed that NfL has a stronger association with retinal thinning compared to P-tau181 levels. Notably, blood plasma biomarkers offer additional diagnostic value for distinguishing high-risk moderate CIND and dementia individuals compared to OCT parameters alone. Further studies in different ethnic groups are required to confirm these findings.

## Supplementary Material

fcae472_Supplementary_Data

## Data Availability

The datasets used and/or analysed during the current study are available from the corresponding authors upon reasonable request.
