# Supplementary material for: Diagnosis of cognitive impairment and dementia: blood plasma and optical coherence tomography
Source: Brain Commun. 2024 Dec 27;7(1):fcae472. doi: 10.1093/braincomms/fcae472 (PMC11694681; doi:10.1093/braincomms/fcae472)
Supplement: fcae472_Supplementary_Data [file fcae472_supplementary_data.docx]

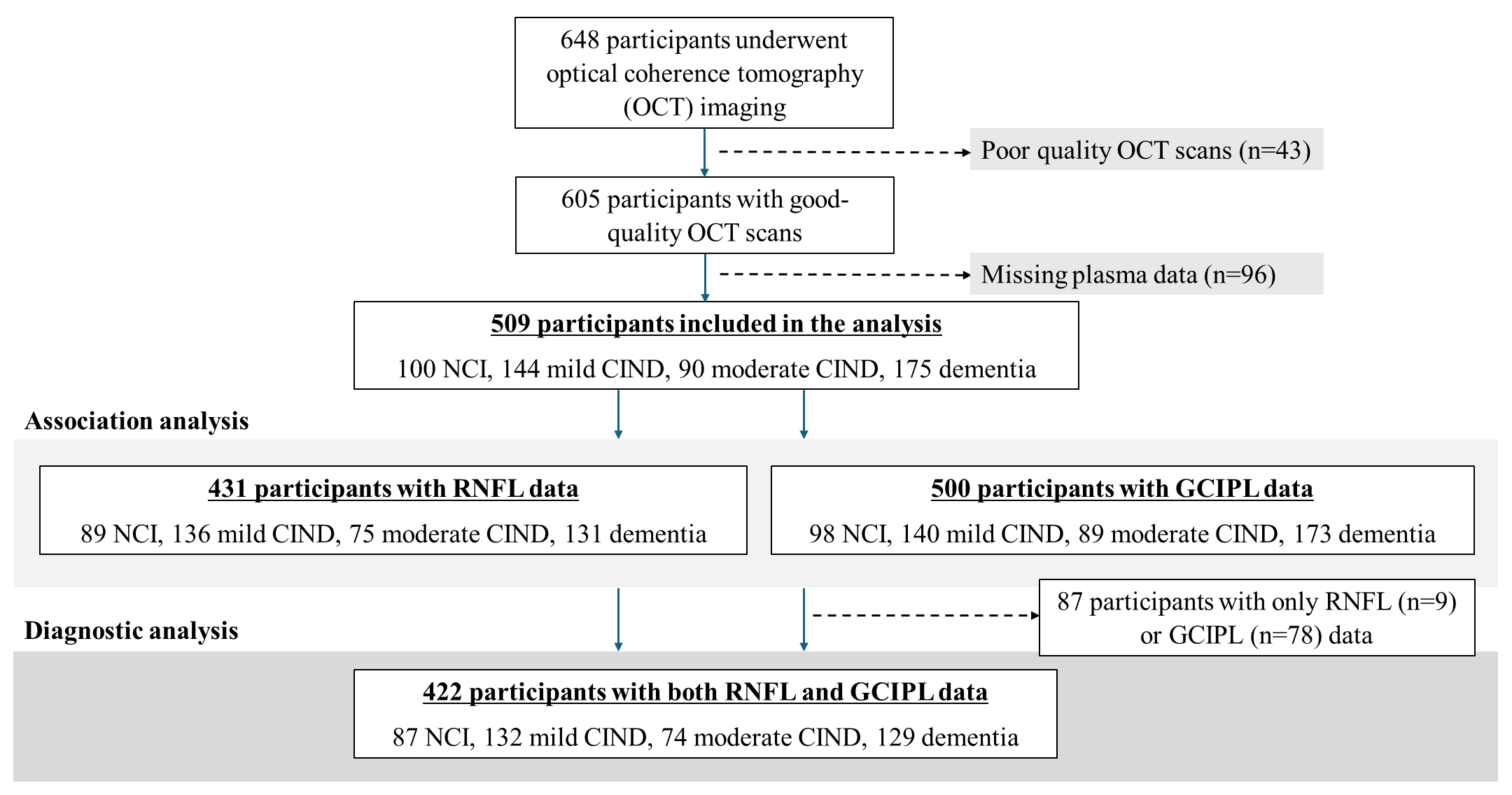
**Supplementary Figure 1.** Flow diagram of the included and excluded participants.

| **Supplementary Table 1. Diagnostic performance for discriminating moderate CIND/dementia from mild CIND/NCI individuals in participants with only normal, healthy eyes (n=192 participants)** | | | | | | |
| --- | --- | --- | --- | --- | --- | --- |
|  | **Area Under ROC** | **95% Confidence Interval** | **Sensitivity (%) at 80% specificity** | **P value** | **P value** | **P value** |
| **OCT** |  |  |  |  |  |  |
| GCIPL | 0.59 | 0.51 to 0.68 | 35% | Reference |  |  |
| RNFL | 0.55 | 0.46 to 0.63 | 23% | **<0.001** |  |  |
| Combined OCT | 0.61 | 0.53 to 0.69 | 31% |  |  | **<0.001** |
| **Plasma** |  |  |  |  |  |  |
| NfL | 0.74 | 0.66 to 0.81 | 56% |  | Reference |  |
| P-tau181 | 0.66 | 0.58 to 0.74 | 36% |  | **<0.001** |  |
| Combined plasma | 0.75 | 0.68 to 0.82 | 60% |  |  | **0.001** |
| **Clinical status** |  |  |  |  |  |  |
| Age | 0.63 | 0.56 to 0.71 | 35% |  |  |  |
| Educational levels | 0.71 | 0.64 to 0.71 | 42% |  |  |  |
| Combined clinical status | 0.73 | 0.66 to 0.80 | 44% |  |  | **<0.001** |
| **Combined models** |  |  |  |  |  |  |
| Combined OCT + Combined plasma + Combined clinical status | 0.81 | 0.75 to 0.87 | 67% |  |  | **Reference** |
| Combined plasma + Combined clinical status | 0.79 | 0.72 to 0.85 | 65% |  |  | 0.029 |
| NfL + Combined clinical status | 0.78 | 0.71 to 0.84 | 50% |  |  | **0.012** |
| Combined OCT + Combined clinical status | 0.75 | 0.68 to 0.82 | 59% |  |  | **<0.001** |
| GCIPL = ganglion cell inner plexiform layer, NCI = no cognitive impairment; NfL= Neurofilament light chain, CIND = cognitive impairment no dementia, P tau 181 = phosphorylated tau at serine 181, RNFL = retinal nerve fiber layer, ROC = receiver operating characteristic curve | | | | | | |
| P value indicates the paired comparisons with the reference parameter using the likelihood ratio test.  Significant P values (<0.017 after Bonferroni correction) are indicated in bold. | | | | | | |
|  | | | | | | |
